# Gold miners augment malaria transmission in indigenous territories of Roraima state, Brazil

**DOI:** 10.1186/s12936-022-04381-6

**Published:** 2022-11-29

**Authors:** Jacqueline de Aguiar Barros, Fabiana Granja, Pedro Pequeno, Paola Marchesini, Maria de Fátima Ferreira da Cruz

**Affiliations:** 1 Núcleo de Controle da Malária/Departamento de Vigilância Epidemiológica/Coordenação Geral de Vigilância em Saúde/SESAU-RR, Roraima, Brazil; 2 Centro de Estudos da Biodiversidade, Universidade Federal de Roraima (UFRR), Roraima, Brazil; 3 Programa de Pós-Graduação em Recursos Naturais, Universidade Federal de Roraima (UFRR), Roraima, Brazil; 4 Coordenação-Geral de Vigilância de Zoonoses e Doenças de Transmissão Vetorial, Departamento de Imunização e Doenças Transmissíveis, Secretaria de Vigilância em Saúde, MS, Brasília/DF, Brazil; 5 Laboratório de Pesquisas em Malária/Malaria Research Laboratory, Instituto Oswaldo Cruz, Fundação Oswaldo Cruz (Fiocruz), Rio de Janeiro, Brazil; 6 Programa de Pós-Graduação em Biodiversidade e Biotecnologia da Rede BIONORTE (PPG-BIONORTE), Roraima, Brazil; 7 Centro de Pesquisa, Diagnóstico e Treinamento em Malária (CPD-Mal)/Reference Center for Malaria in the Extra-Amazonian Region of the Brazilian Ministry of Health, Fiocruz, Rio de Janeiro, Brazil

**Keywords:** Brazilian Amazon, Mining, Malaria, *Plasmodium*, Roraima, Indigenous

## Abstract

**Background:**

Endemic malaria is present in all 15 municipalities of Roraima state, Brazilian Amazonia. Knowledge of epidemiological data of specific populations can guide health policies to formulate effective strategies for integrated control of health-disease care. This study aims to ascertain when, where and who fell ill with malaria in Roraima state from 2010 to 2020.

**Methods:**

This descriptive study was based on statistical secondary surveillance data through the analysis of relationships underlying numbers of cases, hospitalizations and deaths using the Malaria Epidemiological Surveillance Information System, Mortality Information System and Hospitalization Information System.

**Results:**

From 2010 to 2020, there were 138,504 autochthonous cases, 26,158 Venezuelan imported cases, 3765 hospitalizations, and 77 deaths from malaria reported in Roraima. Annual parasitic incidence and the number of hospitalizations showed impressive changes over the period, but without significantly correlating with number of deaths. The proportion of *Plasmodium falciparum* infections had significant shifts throughout this study. Malaria prevalence in indigenous and mining areas has been increasing since 2014.

**Conclusion:**

The presence of miners in indigenous areas is a reality that has been contributing to the increase of malaria cases in Roraima. The need to implement health policies that also meet this contingent is reinforced.

## Background

Malaria is an acute febrile illness caused by the protozoan parasites of the *Plasmodium* genus. Two species are of special relevance to public health: *Plasmodium vivax*, the most spread species worldwide, is predominant in Brazil, while *Plasmodium falciparum,* which predominates in the global setting, is associated with severe malaria, complications of pregnancy and accounts for almost the totality of deaths. In turn, *P. vivax* generally induces uncomplicated malaria, although relapse occurrences may show significant associated morbidity [1, 2]. Transmission occurs through a bite from an infective female *Anopheles* mosquito. In 2020, 241 million cases were estimated globally, and 627,000 deaths were caused by malaria. This represents about 14 million more cases and 69,000 more deaths in 2020 than in 2019 [3]. In Brazil 145,188 cases of malaria were reported in 2020, representing a reduction of 7.8% when compared to 157,452 cases notified in 2019 [4].

Malaria still stands out as a major public health problem in Brazil and around the world, exerting a substantial impact on hospitalizations, complications of childbirth, premature mortality, and work and school absence, despite effective treatment currently available. Besides, malaria is closely related to poverty, exerting a negative impact on the quality of life and socio-economic condition of people leaving in endemic areas [5, 6].

Brazil has a successful background in malaria eradication campaigns from the 1950s and 1960s. However, despite several decades of effort to control infections, the prevalence of malaria in Brazil is still high, with 99% of cases located in the so-called Legal Amazon Region, which comprises the states of Acre, Amazonas, Amapá, Pará, Rondônia, Roraima, Tocantins, Mato Grosso, and Maranhão [5].

During the 1950s, the malaria eradication programme helped to incorporate techniques of epidemiological surveillance to control transmissible diseases throughout the world [7]. The third pillar of the world strategy against malaria 2016–2030 intends to transform malaria surveillance into a basic intervention in the countries where it is endemic [8]. Based on this scenario, Brazil’s Ministry of Health aligned with the Sustained Development Objectives (SDO) of the United Nations launched in 2015, the Plan for Malaria Elimination until 2030 [4].

A crucial step for descriptive epidemiology in identifying the general pattern of an infection and risk groups is the description of the disease in three basic categories: temporal distribution, spatial distribution and distribution according to personal attributes [9].

Roraima is the northernmost state in Brazil and has unique geographic, social and environmental characteristics that make the goal of eliminating malaria even more challenging in its territory. In particular, the movement of people on the international land borders with Venezuela and Guyana increases the risk of imported malaria cases, which are intensified by humanitarian crises, such as the one that occurred recently in Venezuela [10, 11]. In addition, there is currently a recurring threat of illegal gold and diamond mining in the Yanomami and Raposa Serra do Sol Brazilian indigenous areas, respectively. The first is located in Roraima and Amazonas states, along the Venezuela border; it is estimated that there are 20,000 illegal miners in this Yanomami indigenous territory. The second is located on the edge of the crystalline shield of the Guianas, situated along the international political border that is also constantly crossed by miners in both directions [12, 13]. In these indigenous areas, the way environment is altered by open-pit mining, digging hollows and benches throughout the landscape, produces a multiplicity of mosquito breeding places. Besides, shirtless gold miners working during periods of high vectorial activity together with the presence of asymptomatic disease carriers has been contributing to the high incidence of malaria in mining areas. In addition, the great mobility of gold miners also constitutes another risk factor, as it facilitates the renewal of susceptible populations, due to the iterative flow of infected/non-infected people [9].

A set of features, such as landscapes, human presence and vector distribution, intervenes in the epidemiology of malaria. In Northern Brazilian Amazonia, Roraima’s landscapes include 83% of forests and 17% of savannahs. The greater risk for malaria is associated with the prairies, where dense ombrophile forest is the dominant vegetation and in the outskirts of the alluvial forest, followed by a minor risk in the savannah patches. Endemic malaria is present in all the 15 municipalities of the Roraima state, emphasizing the importance of surveillance strategies in all of them [14].

In view of the above, an investigation was carried out for guiding at state level (where problems and solutions really happen) the formulation of effective strategies in the integrated health-disease-care control of this population. To this end, secondary data sources were used because such methodology offers lower costs and faster results when compared to primary data collection, and also because these data are available in government sources, thus offering large arrays of data on a national scale.

The main proposal is to analyse malaria cases in Roraima from 2010 to 2020, to find out when, where and who gets sick from malaria. This period precedes the decade that planned to eliminate vivax malaria by 2035 and falciparum malaria by 2030 in Brazil.

## Methods

An ecological time-series study was based on secondary data concerning the number of cases, hospitalizations and deaths from malaria that occurred in Roraima from 2010 to 2020.

**Fig. 1:**
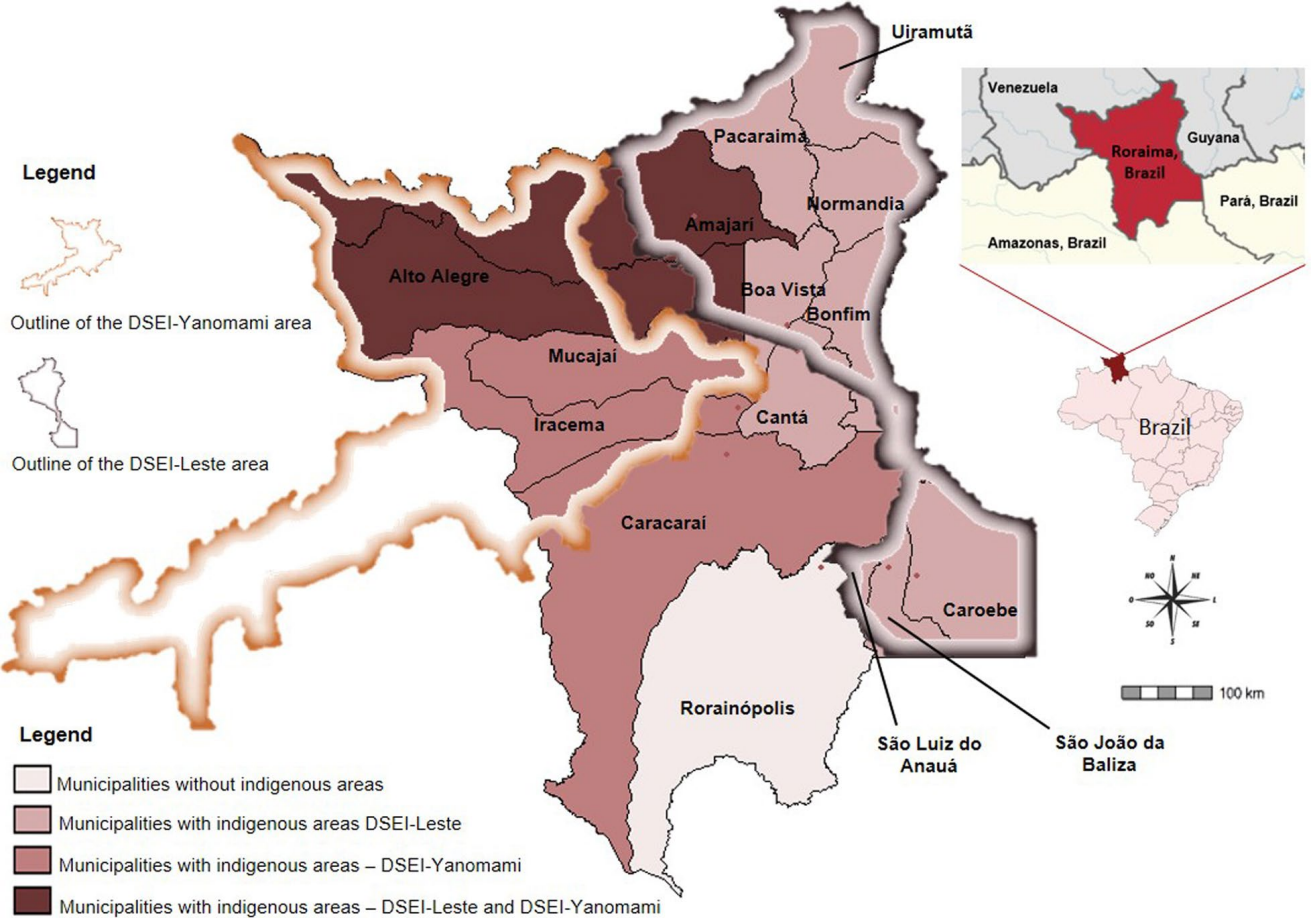
The 15 municipalities of Roraima state showing Leste (DSEI-Leste) and Yanomami (DSEI-Yanomani) Special Indigenous Health Districts (DSEIs)

With a land area of 223,644.527 sq km, the state of Roraima is at the northern tip of Brazil, and borders Venezuela, Guyana and the Brazilian states of Pará and Amazonas. According to the Brazilian Institute of Geography and Statistics (IBGE), the state’s population was estimated at 631,181 inhabitants in 2020. Roraima has 15 municipalities and 104,509,087 sq km of its territory consisting of two main indigenous reservations: the Special Indigenous Health District Yanomami (DSEI-Yanomami) covering five municipalities: Alto Alegre, Amajari, Caracaraí, Iracema, and Mucajaí, and the Special Indigenous Health District Leste (DSEI-Leste) comprising 11 municipalities: Boa Vista, Alto Alegre, Amajari, Bonfim, Cantá, Normandia, Pacaraima, Uiramutã, São João da Baliza, São Luiz do Anauá, and Caroebe. Alto Alegre and Amajari are shared by two indigenous districts. The municipality of Rorainópolis is the only one that has no indigenous area within its territory (Fig. 1).

Malaria is a notifiable disease in Brazil, and its diagnosis and treatment are exclusively available through the Brazilian federal healthcare net of *Sistema Único de Saúde* (SUS)/Health Unic System, including the Health Units of the 15 municipalities of Roraima and indigenous areas. In these municipalities, health facilities are distributed in 253 laboratories: Alto Alegre (36), Amajarí (24), Boa Vista (34), Bonfim (15), Cantá (21), Caracaraí (13), Caroebe (06), Iracema (8), Mucajaí (14), Normandia (17), Pacaraima (21), Rorainópolis (10), São João da Baliza (4), São Luiz (4), and Uiramutã (26). The gold standard for malaria diagnosis is the microscopic examination of a thick blood smear (TBS). The great majority of the cases were diagnosed by microscopy. Rapid diagnostic tests (RDTs) are only realized when the malaria diagnosis by TBS could not be realized in a period of fewer than 24 h and/or in remote places where there is no possibility of installing a microscopy service.

The malaria cases were obtained through Malaria Epidemiological Surveillance Information System (SIVEP-Malaria). The annual parasitic incidence (API) was used to estimate the risk of malaria infection within a given population in a given period of time. The degree of risk of API is expressed as very low risk (< 1 case), low risk (< 10 cases), medium risk (10–50 cases), or high risk (> 50 cases).

The data on mortality and hospitalizations due to malaria were collected in Mortality Information System (SIM) and Hospitalization Information System (SIH), respectively. Both were identified on those systems through codes B50 to B54, according to International Disease Classification (CID-10). The variables concerning parasite species were described as: a) *P vivax*: which could also include results of *Plasmodium malariae*; b) *P. falciparum:* which could also include mixed plasmodial infections; and, c) unspecified malaria when the parasitic species was not identified in the registry.

The following variables were correlated for temporal analysis: site of infection (non-indigenous area, DSEI-Leste and DSEI-Yanomami), API, plasmodial species (*P. falciparum* and *P. vivax*), special areas of infection (indigenous, settlement, urban, mining), Venezuelan imported cases, age group, gender, and race. Each variable, numerical or qualitative, was investigated in the 15 municipalities of Roraima in each year of the investigation period (11 years), resulting in 165 statistical units.

Regarding the temporal trend of hospitalizations throughout the study period, the following variables in each year of the study were considered: parasitic species, gender, race, and age group. In relation to deaths, the comparable variables were parasitic species, gender, race, age group, and nationality.

The statistical analysis was performed through the program R 3.6.3 (R Core Team 2020). A linear model estimated by Generalized Least Squares (GLS) was applied to control the temporal autocorrelation among observations repeated over time (assuming an autoregressive structure of first-order at the municipality level). All the dependent variables were analysed in their original scales, except in falciparum malaria cases which were surveyed in a log scale in order to incorporate the high heteroscedasticity observed in this relation. Graphics were shown only when the associations were statistically significant (p < 0.05).

## Results

During the period 2010 to 2020, 167,968 cases of malaria were notified and 138,504 of them were identified as autochthonous (database consulted on 29 March, 2022). Among the autochthonous cases, 35% (58,597) occurred in indigenous areas: 19,102 in DSEI-Leste and 39,495 in DSEI-Yanomami lands. In the same period, 3,765 hospitalizations and 77 deaths from malaria were reported in Roraima (Table 1).

**Table 1:** Population, malaria autochthonous cases, Roraima annual parasitic incidence (API), malaria cases in indigenous (DSL and DSY), and non-indigenous areas, hospitalizations and deaths in Roraima from 2010 to 2020

Year	Population	Autochthonous cases	API	Non indigenous	DSEI-Leste	DSEI-Yanomami	Hospitalizations	Deaths p = 0.25
			p < 0.001)				p = 0.002	
2010	451,227	19,055	42.23	13,288	2,407	3,360	490	4
2011	460,165	11,860	25.77	7,963	1,603	2,294	312	2
2012	469,524	5,923	12.61	4,375	1,076	472	261	2
2013	488,072	4,828	9.89	3,599	625	604	292	5
2014	496,936	5,713	11.5	4,145	602	966	220	2
2015	505,665	6,176	12.21	3,636	381	2,159	236	4
2016	514,229	5,716	11.12	3,024	368	2,324	254	1
2017	522,636	11,183	21.4	8,647	895	1,641	369	4
2018	576,568	18,346	31.82	11,426	3,199	3,721	497	18
2019	605,761	20,322	33.55	9,025	3,383	7,914	478	16
2020	631,181	29,382	46.55	10,779	4,563	14,040	356	19
Total		138,504	-	79,907	19,102	39,495	3,765	77

Sources: IBGE; SIVEP-MALÁRIA; SIM; SIH

The API presented a significant shift during the decade under study (p < 0.001). In this period, the highest API was recorded in 2010 (42.56/1000 inhabitants), followed by a reduction from 2011 (26.12/1000 inhabitants) to 2013 (10.10/1000 inhabitants), and a new rising trend from 2014 on (11.72/1,000 inhabitants), which lasted until 2020 (46.55/1000 inhabitants) (Table 1).

The non-indigenous and the DSEI-Leste indigenous areas of Roraima have shown an increasing number of malaria cases from 2017 on, with a rate of + 123.81 and + 143.63 in relation to 2016, respectively. DSEI-Yanomami, in turn, presented an increase in the number of malaria cases from 2014 on, at a rate of + 60.26 in comparison to the preceding year (Table 1).

The number of malaria hospitalizations changed significantly over the period of the study (p = 0.002). The year 2010 also presented a higher frequency of hospitalizations due to malaria (490), and a decrease followed afterwards, with rates of 36.33 and 16.34% in 2011 and 2012, respectively. In 2013 there was a net increase of 11.9% in relation to the previous year, followed by a reduction of 24.65% in 2014. As of 2015, the number of hospitalizations began to get higher again, achieving 7.27%, a trend that was kept until 2018, which showed an increase of 34.7% in relation to the previous year. Nevertheless, 2019 was marked by a reduction of 3.82% in comparison to 2018.

The number of malaria deaths did not change significantly over the period 2010–2020 (p = 0.25). Between 2010 and 2017, fewer than six deaths per year were reported. However, in the years 2018 and 2019, 18 and 12 deaths due to malaria were registered, respectively (Table 1).

The number of Venezuelan imported cases increased significantly from 2016 (p = 0.007), reaching a peak in 2018 with 4478 cases. However, in 2019 and 2020 a reduction of 48.95 and 63.68% of Venezuela's imported cases was observed. The incidence of cases in urban and rural areas as well as in settlements declined between 2010 and 2014, but it increased again from then on (p < 0.001). The same was true for the indigenous territories and mining areas, which showed a remarkable rise in cases from 2016 (p = 0.007). When comparing the number of cases that occurred in 2016 and 2020, an increase of 1,090% in indigenous and 75,576% in mining areas was observed (Table 2).

**Table 2:** Imported cases of malaria diagnosed and reported in Roraima, and autochthonous cases in Roraima, according to the special infection area (AEI)

Variables	2010	2011	2012	2013	2014	2015	2016	2017	2018	2019	2020
*Imported*											
Venezuela (p = 0.007)	1406	877	1149	2112	1220	1260	2470	2323	4478	2285	830
Guyana	1347	1351	1296	1619	714	554	772	575	610	433	266
French Guiana	27	16	6	4	6	4	0	5	7	21	6
Suriname	18	20	3	5	5	2	4	5	2	4	5
Other Countries	8	1	8	7	7	3	8	4	7	2	1
Total	2806	2265	2462	3747	1952	1823	3254	2912	5104	2745	1108
*Autochthonous (AEI)*											
Indigenous áreas (p = 0.007)	2559	7105	5929	3959	1554	1238	1578	2606	2726	11,441	18,765
Rural áreas (p < 0.001)	5360	6595	6206	4058	1889	1783	2076	2005	1662	4437	4196
Settlements (p < 0,001)	2447	2909	4513	2065	1092	785	986	564	728	2275	1869
Urban (p < 0.00)	743	1443	2181	1711	1333	959	1008	970	569	1042	1170
Mining (p = 0.007)	11	128	10	7	5	6	4	6	10	851	3027
Total	11,120	18,180	18,839	11,800	5873	4771	5652	6151	5695	20,046	29,027

Source: SIVEP-MALARIA

**Fig. 2:**
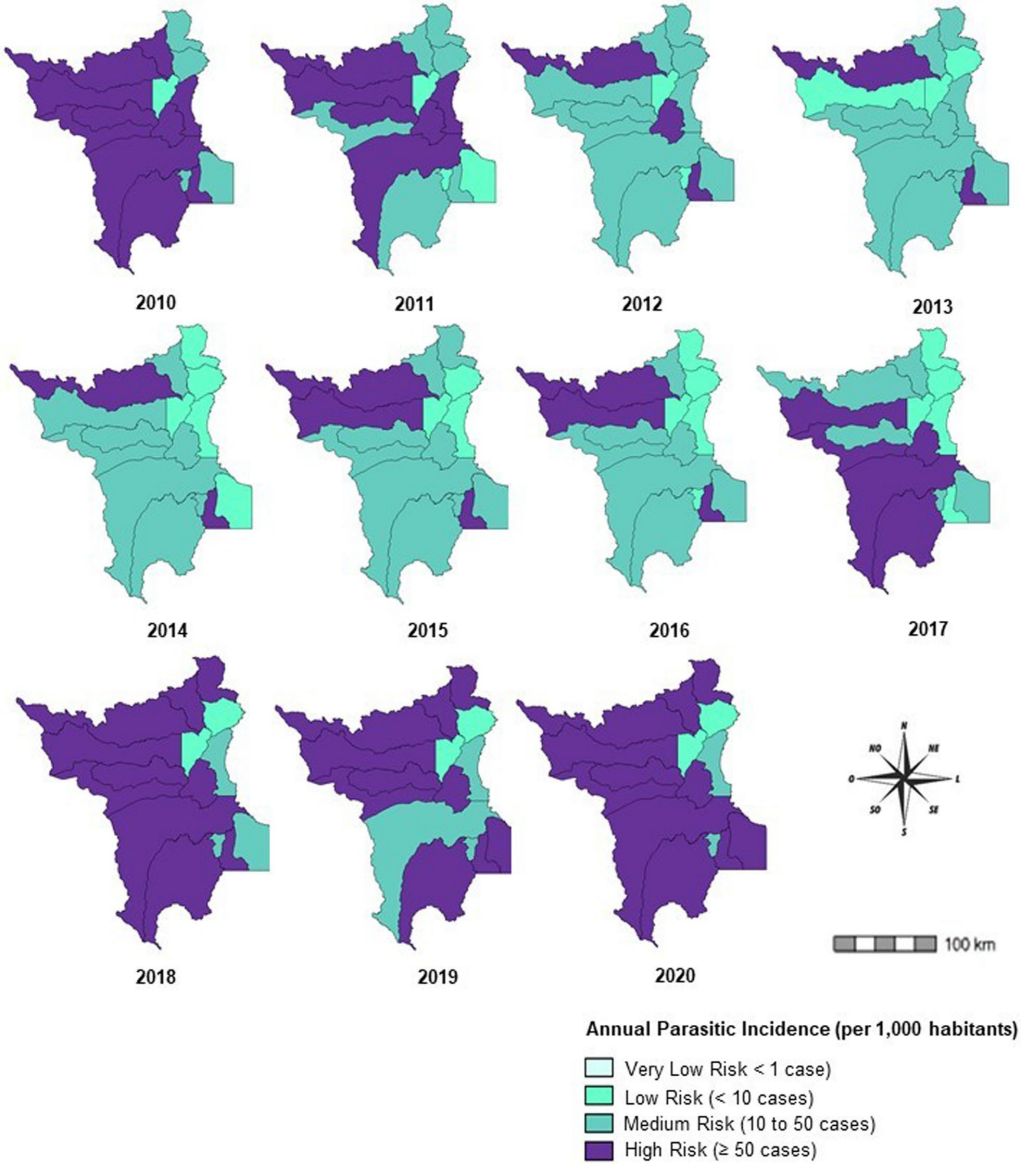
Annual Parasitic Incidence (API) of Roraima state according to the municipality of infection, 2010 to 2020. Sources: SIVEP-MALARIA; IBGE

In 2010, 10 municipalities were classified as high risk (Cantá, Amajari, Alto Alegre, Caracaraí, Bonfim, Iracema, Pacaraima, São João da Baliza, Rorainópolis, Mucajaí). In the following years, the number of high-risk municipalities declined, remaining only the municipalities of Amajari and São João da Baliza. In 2017, however, a new rise of cases occurred, totalling five high-risk municipalities (Cantá, Rorainópolis, Caracaraí, Iracema, Alto Alegre). In 2020, Roraima had again 11 of its municipalities included in the category of high transmission risk (Alto Alegre, Amajari, Pacaraima, Iracema, Uiramutã, Mucajaí, Cantá, Caroebe, São João da Baliza, Caracaraí. Rorainópolis), two classified as medium risk (São Luiz and Bonfim), and two as low risk (Normandia and Boa Vista) (Fig. 2).

Malaria caused by *P. vivax* was predominant during the investigation period. The proportion of *P. falciparum* cases was higher in 2011, reaching 10.67% of the occurrences; the proportion shift of cases caused by *P. falciparum* over the study period was significant (p = 0.01).

In terms of gender, during the decade of the research, the infection rate was greater in men than in women, but statistical analysis has pointed out that the gender ratio showed no significant shift over the period of study (p = 0.35). As for the age group, the cases of malaria seem to present over time a slight increasing trend in the age estimate of most people who contracted malaria, although such evidence is not clear (p > 0.064) (Table 3).

**Table 3:** Roraima state: Autochthonous malaria cases by parasite species, gender and age group, from 2010 to 2020

Variables	2010	2011	2012	2013	2014	2015	2016	2017	2018	2019	2020
*Species*											
*P.vivax*	17,192	10,594	5396	4449	5335	5997	5212	10,961	17,583	19,407	23,276
*P.falciparum*	1863	1266	527	379	378	179	504	222	763	915	6106
% *P.falciparum (p* = *0.01)*	9,78	10,67	8,90	7,85	6,62	2,90	8,82	1,99	4,16	4,50	20,78
*Gender (p = 0.34)*											
Male	11,591	7142	3753	3131	3665	3701	3391	7065	11,562	12,716	17,273
Female	7463	4717	2170	1697	2048	2475	2325	4118	6784	7606	12,109
*Age Group (p > 0.064)*											
0 to 9	5107	3367	1343	995	1234	1785	1612	2075	3880	4755	7644
10 to 19	4785	2853	1451	1069	1317	1525	1427	2593	4164	4536	6700
20 to 39	5821	3578	1923	1649	1836	1672	1596	3599	5979	6918	9895
40 to 59	2713	1720	969	905	1026	937	844	2254	3418	3372	4176
≥ 60	627	339	237	210	300	257	237	661	904	739	967

Source: SIVEP-MALARIA

*Plasmodium vivax* had been doubtlessly the more prevalent parasite species, but even so a tendency of increase in the rate of hospitalizations due to *P. falciparum* seemed to exist over time, although without a statistical meaning. (p = 0.42).

Over the period of research, the average age group of hospitalizations ranged from 20 to 39 years (p = 0.006). In terms of gender, there was no discrepancy among the hospitalization cases over the years (p = 0.71) (Table 4). The correlation between deaths by *P. falciparum* (p = 0.77) and gender (p = 0.18) was not statistically relevant, and the average age groups of malaria deaths were between 20 to 39 years and 40 to 59 years (p = 0.018). In 2019 and 2020, 75 and 73.7% of malaria deaths were caused by *P. vivax*, respectively. In 2018 and 2019, deaths in Venezuelan people represented 61.11 and 37.5%, respectively. In 2020 all deaths occurred in Brazilian people (Table 5).

**Table 4:** Roraima state: Malaria hospitalizations according to parasite species, gender and age group from 2010 to 2020

Variables	2010	2011	2012	2013	2014	2015	2016	2017	2018	2019	2020
*Species*											
*P. falciparum (p* = *0,42)*	85	61	40	64	50	39	61	71	119	91	96
*P. vivax*	376	247	208	221	166	187	185	289	358	379	264
Unspecified	29	4	13	7	4	10	8	9	20	8	16
*Gender (p = 071)*											
Male	175	114	120	93	88	89	100	153	195	176	159
Female	315	198	141	199	132	147	154	216	302	302	217
*Age group*											
0 to 9	105	73	47	48	37	62	46	75	116	95	90
10 to 19	93	64	38	44	25	31	40	77	87	87	54
20 to 39 (p = 0.006)	236	133	113	165	113	99	114	145	221	233	183
40 to 59	38	29	40	29	32	30	42	50	55	52	39
≥ 60	18	13	23	6	13	14	12	22	18	11	10

Source: SIH

**Table 5:** Roraima state: Malaria deaths according to parasite species, sex, nationality and age group from 2010 to 2020

Variables	2010	2011	2012	2013	2014	2015	2016	2017	2018	2019	2020
*Specie*											
*P. falciparum (p* = *0.77)*	1	1	0	2	1	2	0	1	9	2	3
*P. vivax*	2	1	2	1	1	0	0	1	7	12	14
Unspecified	1	0	0	2	0	2	1	2	2	2	2
*Gender (p = 0.18)*											
Male	3	1	1	3	1	2	1	3	10	8	10
Female	1	1	1	2	1	2	0	1	8	8	9
*Nationality*											
Venezuelan	0	1	0	0	0	1	0	1	11	6	0
Brazilian	4	1	2	5	2	3	1	3	7	10	19
*Age Group (p = 0.018)*											
0 to 9	1	1	0	0	0	0	0	0	1	6	8
10 to 19	0	1	0	1	0	1	0	1	3	3	2
20 to 39	2	0	1	1	0	1	0	2	8	4	2
40 to 59	0	0	0	1	2	1	0	1	5	2	3
≥ 60	1	0	1	2	0	1	1	0	1	1	4

Sources: SIM

## Discussion

An overview of malaria epidemiology in the Amazon region from 2003 to 2012 showed the greatest reduction of cases in 2012 when 241,806 cases of malaria were registered, representing a reduction of 60.1% compared to 2005 and 9.1% in relation to 2011 [15]. The malaria cases reduction number observed in Roraima from 2011 to 2013 was also recorded in other Brazilian states of the Amazon Region, mainly in the years 2012 and 2013. This reduction, however, was not homogeneous: the states of Pará, Rondônia and Amazonas presented 69, 40 and 8% of reduction, respectively, in 2013 compared to 2012; meanwhile, in Roraima this reduction rate was 18.64% in 2013 when compared to 2012 [4]. In 2013, the API of 9.89/1000 inhabitants was the lowest identified in the study period. It was the only year in which the Roraima state was classified as low risk for malaria transmission.

Conceivably, this reduction was a result of the actions adopted by the National Programme of Malaria Control (PNCM) of 2005, including: new schedules of *P. falciparum* treatment involving the use of artemisinin-based combination therapy (ACT) and primaquine*;* use of long-lasting insecticidal bed nets; supervision in diagnosis stations; quality control and monitoring of diagnosis performance; use of RDTs; detection systems and epidemic alert; the project of expanded access to prevention and control measures against malaria for Vulnerable Populations of Brazilian Amazon in 2009 (sponsored by the Global Fund to Fight AIDS, Tuberculosis and Malaria), and the Project of Municipal Supporters for Malaria Control in 2012; and, strengthening local team skills in epidemiological investigation, aiming to promote a progressive reduction of malaria cases [15, 16].

It is noteworthy that even considering that the PNCM actions in place in Roraima, API increased 211.67% infection risk in the time span 2014 to 2020, diverging from the reduction of malaria cases observed in the rest of Brazil in the same period. In addition, data from the Ministry of Health pointed to a decline of 19.1% from 2017 to 2020 [4]. In fact, the distribution over time of malaria cases in Roraima from 2010 to 2020 presented a significant variation over the decade, although during the same period API has shown a medium degree of infection risk (API 10–49.9/1000 inhabitants). However, after the reduction in the number of malaria cases in 2012 and 2013 in Roraima, the cases increased by 18.13% in 2014, specifically in the DSY areas, due to the return of illegal mining activities, mainly on the banks of the Uraricoera, Mucajaí and Couto de Magalhães rivers. In reality, mining activities on the banks of the Uraricoera river already existed from 1987 to 1989 in such a way that up to 2,003 mining rafts were counted near the indigenous community of Waikás. These mining operations were shut down in 1991 after the conclusion of the indigenous territories’ boundary demarcation. But, in 2010 new gold mining rafts returned to the Uraricoera. Despite the efforts of local leaders, gold miners refused to leave the region, alleging that mining activity was the only source of income for their families. By the end of 2016, 133 open-pit gold-mines were identified in the Yanomami area, and were opened with greater momentum from 2018 [13, 17].

The scenario of increasing malaria cases triggered by illegal gold mining in the indigenous Yanomami areas and the resulting impact on the local health system has established several meetings of teams involved with malaria control in the Roraima DSEIs municipalities and Ministry of Health/SESAI representatives, in an effort to find effective solutions.

In 2018 a proposal of registering gold miners inside Yanomami lands in the SIVEP-Malaria platform was put forward, in order to allow the stratification of transmission data by origin in indigenous areas with or without mining activities, as control actions adopted in each case are distinct. This record from 2019 in Roraima, showed the increase in the number of autochthonous cases of malaria in mining areas. However, the registration of malaria cases from mining locations is far from reality, reinforcing the need for professional training for malaria notifiers, as well as the investigation of the likely source of infection of the reported cases. It is worth noting that the illegality of gold mining activities in Roraima hinders state efforts to control the disease in the municipalities and DSEI-Yanomami, in terms of safety and logistics issues.

The access routes to the mining areas are mainly through the rivers and forest areas of the municipalities of Alto Alegre, Amajari, Mucajaí, Caracaraí, and Iracema, or by plane through clandestine hidden airstrips in rural areas. The API spatial analysis of these municipalities during the study period showed that Amajari and Alto Alegre presented the largest periods under high infection risk. Amajari presented a high infection risk over the whole decade, except for 2017 when it presented a medium risk. Alto Alegre, in turn, was under high risk of infection for seven years, except for 2012 and 2014, when it showed medium risk, and in 2013 low risk.

The pressure of illegal mining is greater in the Yanomami indigenous areas, however, more recently, this activity has also been identified in the Raposa Serra do Sol Indigenous Land, in the DSEI-Leste, located in the municipalities of Normandia, Pacaraima and Uiramutã, between the Tacutu, Maú, Surumu, and Miang rivers and on the Venezuelan border [18]. A survey carried out in 2020 showed the existence of illegal diamond mining in the municipalities of Uiramutã and Pacaraima [13]. During the study period, these municipalities showed a high risk of malaria transmission from 2018, which remained until 2020.

According to the World Health Organization, malaria cases in the Americas fell from 894,000 in 2019 to 653,000 cases in 2020. Part of this reduction was attributed to movement restrictions during the COVID-19 pandemic and to the lack of fuel affecting mining activities. Such restrictions could also have affected access to care and case detection [3]. The malaria scenario in Roraima during the COVID-19 pandemic was different, with a 44% increase of autochthonous malaria cases in 2020 when compared to 2019. Interestingly, in 2020, there was also a 30% increase in mining activities in Yanomami indigenous land, mainly in the Waikas and Kanayanau regions. The new illegal mining centres were located mainly in the channels of the Uraricoera river, which concentrates 52% of the total area of illegal mining in the Yanomami indigenous land. Illegal mining was also identified on the banks of the Parima, Mucajaí, Couto de Magalhães, and Catrimani rivers [17].

Malaria control in the gold mines of Roraima is a major challenge. In addition to the difficulty in controlling the vector, mining is illegal and is carried out in areas of difficult access, making timely diagnosis and treatment considerably difficult [19]. Another problem in mining areas is that people infected with malaria often self-medicate with erratic regimens, often using just a dose called ‘incubator’ to quickly eliminate symptoms and return to mining. These non-curative underdoses favour parasites resistant to anti-malarials [20, 21]. In addition, miners use drugs of dubious quality, such as Artecom® (artemisinin-based medication), which is not registered by a drug regulatory authority or by the WHO prequalified programme and is therefore illegal in French Guiana and neighbouring countries [22].

Malaria control in illegal miners goes beyond the domain of public health, and it is necessary to include other government bodies in the debate on gold mining in indigenous lands, which as in any other economic activity must take principles of sustainability, preservation of biodiversity and guarantee the cultural and social rights of indigenous peoples to ensure social wellbeing and health of indigenous and non-indigenous populations.

In 1961, the Bolivarian Republic of Venezuela (hereinafter called Venezuela) was the first country certified by WHO to eradicate malaria; nevertheless, as of 2012 the country’s situation turned alarmingly. Economic collapse in Venezuela led to a lack of anti-malarial medication and the failure of other control measures, resulting in a rise in case numbers, both in endemic and non-endemic regions, affecting neighbouring countries with imported malaria cases, including infections due to *P. falciparum* [23]. The economic crisis has driven many people to illegal gold mining, where they contract malaria and spread the disease when returning home. Cases of imported malaria proceeded mainly from Bolivar and Amazonas, Venezuelan states bordering Brazil.

On the Brazilian side, the Pacaraima municipality has a population of 11,667 inhabitants, including the indigenous population. The seat of the municipality, the only non-indigenous area in the municipality, is located at an average altitude of 900 m and has vegetation cover of steppe savannah, thus presenting a negligible risk for malaria transmission. However, the indigenous areas of São Marcos, located alongside highway BR-174, offers favourable environmental conditions for the mosquito vector. Venezuelan immigration has contributed to the recrudescence of autochthonous cases in these native communities because they serve as shelters for refugees when they move to the capital, Boa Vista, along the BR-174, and in 2018 to 2020, the municipality of Pacaraima presented a high risk of malaria infection. The biggest reduction in 2020 can be explained by the Venezuelan border closing during the period of the Covid-19 pandemic. On the other hand, an increase in autochthonous cases, mainly in indigenous localities of DSEI-Leste, located in the municipality of Pacaraima, was recorded.

Indigenous reserves occupy 70% of areas in the municipalities of Normandia, Uiramutã, Alto Alegre, Pacaraima, and Iracema. In these municipalities, malaria infections are concentrated in the indigenous area, with little or no transmission in non-indigenous areas.

The measures of malaria prevention and control in indigenous areas are also a challenge to public health, due among other reasons to environmental changes and to nomadic behaviour and cultural characteristics such as hunting, fishing, farming, and bathing in rivers and streams [5]. Some studies point out that in the Amazon, the risk of indigenous people getting sick from malaria is twice that of non-indigenous people. These studies indicate that in the period 2003 to 2012 the epidemic municipalities were characterized by having indigenous populations, settlements, gold mining, and international borders [6, 15, 24].

Timely diagnosis and treatment have a greater impact on the control of malaria caused by *P. falciparum*, since *P. vivax* infections present gametocytes (the infectious mosquito stage) from the first days of infection; in turn, *P. falciparum* gametocytes are found in the bloodstream only after seven days of infection [5]. Thus, a significant percentage rise of infections due to *P. falciparum,* reported over the observation period, highlights the lack of access to opportune treatment, mainly in gold miners’ malaria cases. If not timely treated, malaria can evolve to its severe forms, causing hospitalizations and deaths. The percentage of hospitalizations due to malaria, not only in Brazil but also in endemic areas of the world, is directly proportional to the provision of timely diagnosis and adequate treatment.

*Plasmodium vivax* infections have been increasingly associated with an important, multisystemic impact on individual health, mainly in the presence of co-morbidities [25]. Vivax malaria cases may also develop into more serious forms of the disease, either because of inexperienced outpatient care, late diagnosis, inadequate/incomplete treatment, or even drug resistance [26, 27].

The number of deaths caused by malaria in Roraima can be considered high when compared to national data. While from 2000 to 2017 there was a progressive reduction of deaths nationwide (from 245 to 34), in Roraima 18 deaths caused by malaria were reported in 2018 alone, although from 2010 to 2017 the mortality rate averaged three deaths per year, with up to 24 deaths within a period of eight years. Besides this increase, in 2019 deaths due to malaria in Roraima made up 46% of the total reported for Legal Amazon [4]. These data are probably due to a larger number of imported cases from Venezuela (11 deaths) and the increase of malaria infections in gold mining areas among Brazilians (7 deaths) in 2018. In 2019, 16 deaths, six in Venezuelans and 10 in Brazilians were reported in Roraima. These figures exemplify how difficult is timely diagnosis and treatment in Venezuela and in mining areas.

This study, like any study based on secondary data, may have some limitations related to possible underreporting, incompleteness and inadequate registration.

## Conclusions

Malaria remains a serious health problem in Roraima, and two factors are responsible for the increase of malaria cases in the decade of the study.

The first, related to cases imported from Venezuela, exemplifies the challenge of controlling malaria in border areas, making it essential to maintain and strengthen surveillance for early identification of any change in malaria epidemiological patterns of importation or re-introduction in border localities.

The second is related to illegal mining in indigenous areas that returned in 2010 and with greater impetus from 2018 onwards. In 2020, during the Covid-19 pandemic, the pressure of this activity was more intense in the Yanomami indigenous area but also occurred in the indigenous region of Raposa Serra do Sol. Malaria control challenges in indigenous areas include the cultural diversity of indigenous peoples, housing (that sometimes does not allow for vector control) and the presence of illegal mining. It is mandatory that new health policies include this contingent, providing resources and strategies in order to reduce infections.

Actions to control malaria in illegal mining transcend the health area for formulation of public policies and coordination with other institutions at municipal, state and federal levels is required, especially in relation to strategies to increase access to diagnosis and timely treatment. Health education for this population should also be considered, on the importance of carrying out complete treatment to avoid an increase in hospitalizations and deaths on taking medication of dubious origin to treat malaria and without adequate diagnosis, to the importance of monitoring molecular markers of resistance to anti-malarials in Roraima, in order to control the spread of *P. falciparum* and *P. vivax* parasites resistant to anti-malarials currently available.

## Abbreviations

SDO: Sustained Development Objectives
IBGE: Brazilian Institute of Geography and Statistics
DSEI-Yanomami: Special Indigenous Health District Yanomami
DSEI-Leste: Special Indigenous Health District Leste
SIVEP-Malaria: Malaria Epidemiological Surveillance Information System
SIM: Mortality Information System
SIH: Hospitalization Information System
CID-10: International Disease Classification
API: Annual Parasitic Incidence
GLS: Generalized Least Squares
PNCM: National Programme of Malaria Control
SUS: Health Unic System
ACT: Artemisinin combination therapy
WHO: World Health Organization
